# MALDI Mass Spectrometry Imaging of Lipids and Gene Expression Reveals Differences in Fatty Acid Metabolism between Follicular Compartments in Porcine Ovaries

**DOI:** 10.3390/biology4010216

**Published:** 2015-03-06

**Authors:** Svetlana Uzbekova, Sebastien Elis, Ana-Paula Teixeira-Gomes, Alice Desmarchais, Virginie Maillard, Valerie Labas

**Affiliations:** 1 INRA, UMR INRA 85-CNRS 7247-Université de Tours-IFCE, Physiologie de la Reproduction et des Comportements, F-37540 Nouzilly, France; E-Mails: sebastien.elis@tours.inra.fr (S.E.); alice.desmarchais@tours.inra.fr (A.D.); virginie.maillard@tours.inra.fr (V.M.); valerie.labas@tours.inra.fr (V.L.); 2 INRA, Plate-Forme d’Analyse Intégrative des Biomolécules, Laboratoire de Spectrométrie de Masse, F-37380 Nouzilly, France; E-Mail: Ana-Paula.Teixeira@tours.inra.fr; 3 INRA, UMR 1282 Infectiologie et Santé Publique, F-37380 Nouzilly, France

**Keywords:** lipids, ovary, MALDI MS imaging, oocyte, follicular cells, fatty acid metabolism, gene expression, porcine

## Abstract

In mammals, oocytes develop inside the ovarian follicles; this process is strongly supported by the surrounding follicular environment consisting of cumulus, granulosa and theca cells, and follicular fluid. In the antral follicle, the final stages of oogenesis require large amounts of energy that is produced by follicular cells from substrates including glucose, amino acids and fatty acids (FAs). Since lipid metabolism plays an important role in acquiring oocyte developmental competence, the aim of this study was to investigate site-specificity of lipid metabolism in ovaries by comparing lipid profiles and expression of FA metabolism-related genes in different ovarian compartments. Using MALDI Mass Spectrometry Imaging, images of porcine ovary sections were reconstructed from lipid ion signals for the first time. Cluster analysis of ion spectra revealed differences in spatial distribution of lipid species among ovarian compartments, notably between the follicles and interstitial tissue. Inside the follicles analysis differentiated follicular fluid, granulosa, theca and the oocyte-cumulus complex. Moreover, by transcript quantification using real time PCR, we showed that expression of five key genes in FA metabolism significantly varied between somatic follicular cells (theca, granulosa and cumulus) and the oocyte. In conclusion, lipid metabolism differs between ovarian and follicular compartments.

## 1. Introduction

Successful reproduction is largely dependent on oocyte developmental competence, that is, the ability of the female gamete, the oocyte, to undergo fertilization and subsequent embryo development. In mammals, such competence is acquired inside the follicle in the ovary through folliculogenesis [1]. The ovarian follicle grows from the primordial stage, where an oocyte is surrounded with only a single layer of follicular cells, to the antral follicle that is characterized by an antrum cavity filled with follicular fluid (FF) and specialized follicular cells: steroidogenic wall theca cells, granulosa cells (GC) and their derivate cumulus cells (CC), which surround an oocyte and form the oocyte-cumulus complex (OCC). Each cycle, only a few follicles from the antral cohort are selected for final growth and maturation and thus progress to the preovulatory stage. Most of the remaining follicles are eliminated by atresia. The mechanisms of oocyte selection are not fully understood. However, oocyte growth strongly depends on a tight metabolic relationship between an oocyte and its ovarian follicular environment [2].

Ovarian cells can use multiple metabolic substrates including glucose, amino acids and lipids; the flexibility of the metabolic pathways available for energy production during oocyte growth and maturation seems to be a key to high developmental competence of the oocyte [2,3]. Folliculogenesis and final oocyte maturation are regulated at the endocrine and paracrine levels and are strongly influenced by dietary fat supplementation in cows [4] and humans [5]. Although glucose metabolism has largely been studied in ovarian follicular cells and is considered essential in determining oocyte developmental potential (for a review, see [6]), intrafollicular lipid metabolism has been revealed to be particularly important in farm species (cattle, sheep, pigs) with oocytes that contain relatively high concentrations of lipids compared with humans [7].

Apart from energy supply, intracellular lipids have a critical role in biological membrane functions, cell-to-cell interaction, cell proliferation, transport, and regulation of enzyme activity. Lipids of different classes, such as fatty acids (FAs), glycerolipids (mono-, bi-, and tri-acylglycerols), glycerophospholipids, sphingolipids, sterol and sterol esters, *etc.* and their derivatives, are essential components in different endocrine and cell signaling pathways [8]. Reproductive processes are strongly regulated by FAs through a variety of mechanisms (for a review, see [9]). Thus, FAs provide the precursors for prostaglandin synthesis and can modulate the expression patterns of many key enzymes involved in both prostaglandin and steroid metabolism, which play a crucial role in female reproduction. In addition, mitochondrial β-oxidation of free FAs that are either released from triacylglycerols (TAGs) through lipolysis or synthesized *de novo*, is important for energy supply for ovarian cells which can store lipids and possess lipogenic and lipolytic properties [10,11,12]. Indeed, recent studies have demonstrated that FA oxidation in the oocyte is required for the completion of oocyte meiotic maturation, the final step of oocyte development before fertilization, in different species including cattle, pigs and mice [13]. Moreover, CC can also accumulate lipids from the OCC environment [14] and thus protect the oocyte from the lipotoxity induced by excessive FAs [15]. Therefore, lipid metabolism is of great importance for oocyte developmental competence.

Lipid metabolism has been shown to be regulated by a set of FA metabolism-related genes (for a review, see [16]). Among the key factors of FA metabolism are acetyl coenzyme A carboxylase (*ACACA*) involved in FA synthesis, carnitine palmitoyl transferase 1 (CPT1) involved in FA β-oxidation, FA-binding proteins (FABP) involved in FA transport in the cell, thrombospondin receptor alias FA translocase CD36 involved in the FA entry in the cell, and perilipins (PLIN), which are involved in lipolysis and are located in the periphery of lipid droplets. Free FAs may occur through *de novo* synthesis using acetyl-CoA, through lipolysis of TAGs in lipid droplets, or through importation from the follicular environment via FA membrane transporters such as CD36. Inside the cell, FAs may be transported by FABPs and can either be transformed and stored in lipid droplets or directed to mitochondria using CPT1 where FAs are metabolized through FA oxidation, thus producing ATP.

Although lipid composition of FF, oocytes and surrounding CC has already been reported in humans, cattle and pigs [17,18,19,20,21,22,23], spatial distribution of lipid species throughout the ovary has never been studied. Matrix Assisted Laser Desorption/Ionization Mass Spectrometry Imaging (MALDI MSI) is a new powerful method for analyzing the spatial distribution of small molecules such as proteins, peptides, lipids, drugs and metabolites in tissues of interest; these specific molecules can be clearly assigned to their cellular origin (for a review, see [24,25]). Moreover, MALDI MSI is particularly promising in clinical research because it has successfully been used for the discovery of candidate biomarkers in cancer therapy [26,27]. MALDI MSI has also been used for direct molecular profiling and imaging of both male and female reproductive tissues such as murine uterus, epididymis and seminiferous tubules [28].

MALDI MSI analyses of ovaries have been performed only for cancer research [29]. Taking into account the growing interest in the role of lipid metabolism in ovarian folliculogenesis and oocyte developmental competence [16,30,31], we aimed to analyze both the spatial distribution of lipid species throughout the ovary and the expression of FA metabolism-related genes in different follicular compartments to identify the cells that are particularly involved in FA metabolism in ovaries. Porcine ovary was used as a model because of its particular lipid metabolism, notably its very high oocyte lipid level [32]. In our study, for the first time, we analyzed the spatial distribution of lipids throughout the ovary by using MALDI MSI and quantified the expression of a set of FA metabolism-related genes in different ovarian cell types.

## 2. Experimental Section

### 2.1. Ethics

Porcine ovaries were obtained from a local commercial slaughterhouse; no experiments on live animals were performed.

### 2.2. Reagents

All reagents were purchased from Sigma (Saint-Quentin Fallavier, France) unless otherwise stated.

### 2.3. Tissue Collection and Preparation

Whole ovaries from slaughtered 9–10 month Large White gilt pigs in the follicular stage of the estrus cycle (*n* = 3) were snap frozen in vapor of liquid nitrogen and kept at −80 °C until use. Before tissue section the ovary was placed at −20 °C for 1 h in a microtome chamber. Ovary sections were cut using a Cryo-Star HM 560 cryostat (Microm, Francheville, France) with a specimen holder chilled at −18 °C. The 14 µm-thick sections were thaw-mounted onto conductive Indium Tin Oxide (ITO)-coated microscope slides (Bruker Daltonics, Wissembourg, France). For external mass calibration, 0.5 μL of peptide calibration standard II (Bruker Daltonics, Wissembourg, France) was placed near the tissue section and mixed (1:1 v/v) with the matrix employed for the MALDI MSI.

Ovary sections were scanned before matrix deposition using a histology slide scanner (Opticlab H850 scanner, Plustek, Ahrensburg, Germany). The histological image was used for the teaching point step to superpose histological and molecular images.

### 2.4. Matrix Coating and MALDI MSI

**Figure 1:**
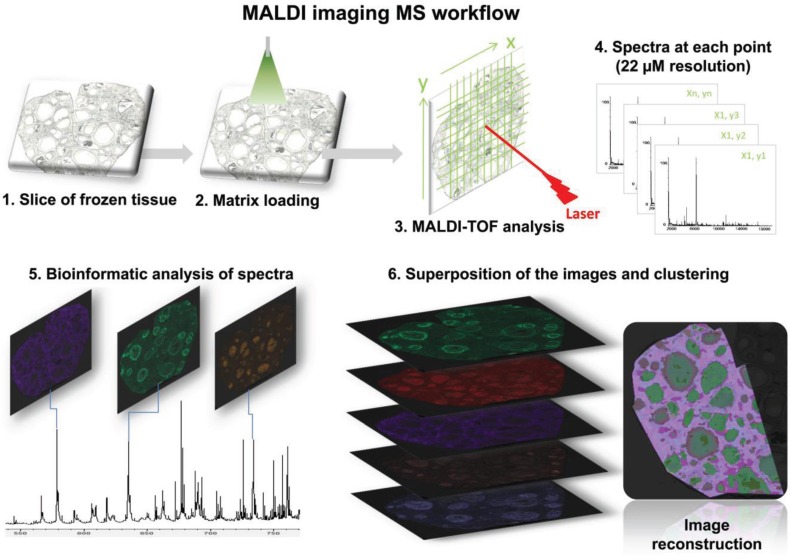
Schematic representation of MALDI Mass Spectrometry Imaging (MSI) of lipids performed on fresh frozen sections of *Sus scrofa* gilt ovaries. Cryostat ovary section was transferred onto an ITO glass slide (**1**). The slide was then covered with α-CHCA or DHB (**2**). After matrix drying, the slide was introduced into the MALDI-TOF mass spectrometer for analysis (**3**). The laser scans through a set of preselected locations with a spatial resolution set at 50, 35 or 22 µm (**4**) generated mass spectra of lipids at each point (**5**). After normalization, ion density maps were created (**6**) for the ions, which were observed from the representative mass spectra showing the major molecular species (skyline projection spectra). Hierarchical cluster analysis displaying discriminatory signals and creating groups of related molecules based on their classification allowed the reconstruction of a molecular image (**7**).

The MALDI MSI workflow is presented in Figure 1. The matrix was coated using an Image Prep device (Bruker Daltonik GmbH, Bremen, Germany) using one of three methods: spraying with α-cyano-4-hydroxycinnamic (CHCA) acid matrix at 7 mg/mL in 60:40 acetonitrile/H_2_O, 0.2% trifluoroacetic acid (TFA); spraying with 2,5-dihydroxybenzoic acid (DHB), in 50:50 acetonitrile/H2O, 0.2% TFA; or using the manufacturer’s standard protocol. The slides were placed in a desiccator for 1 h before MALDI MSI analysis. Spectra were acquired using an UltrafleXtrem MALDI-TOF/TOF instrument (Bruker Daltonik GmbH, Bremen, Germany) equipped with a Smartbeam laser (Nd:YAG, 355 nm) at 2 kHz laser repetition rate at the “small focus” setting. The FlexControl 3.0 software (Bruker Daltonics, Bremen, Germany) was used to control the instrument. Spectra were obtained in both positive and negative reflector ion mode in the 200–1200 m/z range. The accelerating voltage was set to 25 kV. Lipid mass spectra were acquired with spatial resolution set at 50, 35 or 22 µm. At 22 µm, for each pixel, 500 spectra were collected as a sum of 50 consecutive laser shots in 10 random walk shot steps. External calibration was performed using Bruker Peptide Calibration Standard. Raw spectra were analyzed with FlexImaging 4.0 software (Bruker Daltonik GmbH) after baseline subtraction and Root Mean Square (RMS) normalization. Ion density maps were created for ions observed from the skyline projection spectrum. Masses were manually selected with a mass accuracy set to ±0.1% (manual peak picking). Anatomical regions of interest (ROIs) were manually defined by using both the histological image and MSI data. For signals located in a ROI, a mass spectrum associated with a high-intensity pixel was opened with FlexAnalysis 3.4. Mass spectra were smoothed using the Savitzky-Golay algorithm with the following parameters: 0.2 m/z, one cycle and baseline subtracted using the Top Hat algorithm. For the whole acquisition region and ROIs, hierarchical cluster analyses were performed using FlexImaging software. Three ovaries from different gilts were sectioned. MSI was performed on different sections in positive and negative modes. MSI images at 22 µm resolution were used for cluster analysis of individual follicles.

### 2.5. MALDI MSI Lipid Profiling Data Analysis

After hierarchical cluster analysis on ROIs targeted at different follicles, twelve MALDI spectra were extracted from characteristic compartments of individual follicles (theca, granulosa, OCC and FF) according to the morphology in the histological scan images. For an individual follicle, presenting all compartments, a total of 48 spectra were generated for positive and negative mode MSI analysis. Each spectrum was converted to .txt files using FlexAnalysis 3.4 software and integrated in Progenesis MALDI version 1.2 software (NonLinear Dynamics, Newcastle upon Tyne, UK). All spectra were processed with baseline subtraction (Top Hat filter 60), denoising (Noise filter 4) and spectra alignment steps to maximize correlations. Automatic peak detection was applied to the reference spectrum (a weighted average of all experimental spectra) with a threshold fixed at 200 counts. Thus, peaks were detected with a signal:background ratio > 5. Normalization of peak height was performed using the Total Ionic Count (TIC) in order to display and compare all spectra on the same scale. The repeatability of analysis of different follicular compartments linked directly to the spectrometer process was evaluated by a coefficient of variation (CV) on the 20 biggest peaks selected on mean normalized peak height. CV was calculated using normalized peak height of the 12 spectra for one experiment (negative mode). Mean CV values did not exceed 35% for theca, 30% for granulosa, 45% for OCC and 30% for FF. To characterize differences in peak intensity between the follicular cell types, the mean normalized peak height intensity values were subjected to one-way analysis of variance (ANOVA) followed with Fisher’s *post hoc* test. Details of detected m/z peaks are provided in the Supplementary data (Table S1).

### 2.6. RNA Analysis: Quantification of Gene Expression

Small follicles (2–5 mm) from the ovaries of gilts in the follicular stage of the estrus cycle were dissected to isolate GC by gentle scraping of the inside of the follicles. Theca cells were collected after GC removal and three washes of the inside of the follicle with PBS 1X. GC and theca samples were recovered from 12 individual follicles of six different gilts. OCC were recovered from the remaining follicles of the same ovaries, and the oocytes were stripped of CC under a binocular microscope by repetitive aspiration—ejection using a micropipette. Cells were precipitated by centrifugation (3000 g, 5 min) and kept at −80 °C in TRIzol reagent (Life Technologies, Saint Aubin, France). Total RNA extraction was performed using the TRIzol purification system according to the manufacturer’s instructions (*n* = 10 for theca, *n* = 8 for GC, *n* = 12 for CC and *n* = 8 for the oocytes). Total RNA from 25 oocytes or 100 ng of total RNA (from Th, GC and CC) were subjected to Reverse Transcription (RT) and real time Polymerase Chain Reaction (PCR) as previously described [33]. Real time PCR reactions were carried out in 20 μL containing 1× qPCR Mastermix Plus for SYBR Green I (BioRad, Marnes-la-Coquette, France), specific primers (Table 1) at a final concentration of 150 nM, and 5 μL of the RT reaction diluted 1:10 (equivalent to 2.5 ng cDNA) for Th, GC and CC or 1:30 for oocyte (cDNA equivalent to 0.2 oocyte). Real time PCR quantification of gene expression was performed using CFX96 (BioRad) with all the samples in duplicate. The efficiency of the primers and standard curve for each gene was deduced from serial dilutions of the corresponding cDNA fragment obtained as a template.

The geometric mean of two housekeeping genes (*RPL19* and *RPS9*) was used to normalize gene expression. The relative amounts of gene transcripts (R) were calculated according to the equation:

$$\text{R}\;=\;\frac{\left(E_{gene}^{-Ct\;gene}\right)}{\left(\text{geometric mean}\;\left(E_{RPS9}^{-Ct\;RPS9};\;E_{RPL19}^{-Ct\;RPL19}\right)\right)}$$

where Ct is a cycle threshold and E is PCR efficiency for each primer pair (Table 1). Normalized values of relative expression were compared by one-way ANOVA with Fisher’s *post hoc* test (Statview version 5.0, SAS Institute, Inc., Cary, NC, USA.). Differences were considered significant when *p* < 0.05.

**Table 1:** Primers used for real-time PCR gene expression analysis.

Gene	Primer	Séquence 5'–3'	Accession Number	Amplicon (bp)	Gene Product	Primers Efficiency
***ACACA***	fw	TGGAGAAACAGCTGACGGAG	EU168399.1	196	Acetyl coenzyme A carboxylase	
	rev	GAGAGGATCCGGACGACTTC				1.99
***CD36***	fw	TTGGCCTATGAACCGTTTACT	NM_001044622.1	249	CD36 molecule (thrombospondin receptor)	
	rev	CGTTCTGAAGTTGCCAAGCA				1.91
***CPTA1***	fw	ACGTGTCGAAAGAAGGAGGT	NM_001129805.1	138	Carnitine palmitoyl transferase 1 A	
	rev	ACATCCCAAAGAGCATATCGTA				1.95
***FABP5***	fw	ATGGGTGCAATGGCCAAAC	NM_001039746.2	221	Fatty acid binding protein 5	
	rev	GAACCACCACCATGGCATAGA				1.91
***PLIN2***	fw	GTGCCACACCCTCGTG	NM_214200.2	242	Perilipin 2 (alias Adipophilin)	2.00
	rev	AGGGACCTACCAGCCAGTT				
***RPL19***	fw	ATGAAATCGCCAACGCCAAC	AF435591.1	173	Ribosomal protein L19	1.96
	rev	AGCATTGGCAGTACCCTTCC				
***RPS9***	fw	GTGCTGGGGTGCTCTTTAGT	XM_005664825.1	160	Ribosomal protein S9	1.92
	rev	GAGCCCATATTCGCCGATCA				

## 3. Results and Discussion

### 3.1. MALDI MSI Analysis of Porcine Ovarian Sections

The molecular specificity and sensitivity of MS was used in this study for direct mapping and imaging of lipids present in porcine ovarian tissue (Figure 1). Spatial distribution of numerous lipid species throughout ovarian sections was observed using both DHB and CHCA matrices.

We have chosen to investigate the differential spatial distribution of lipids in ovarian compartments and particularly inside the individual follicles by using MALDI MSI with α-CHCA matrix at the highest resolution (22 µm).

#### 3.1.1. Ovarian Lipid Distribution

**Figure 2:**
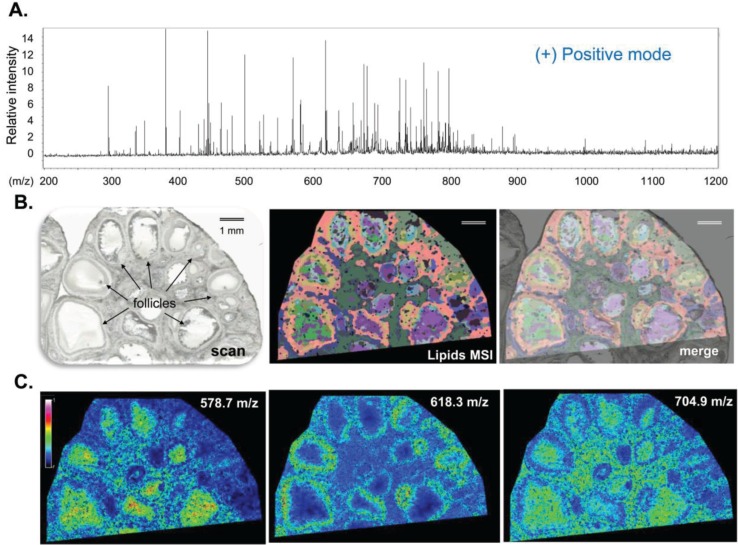
High-resolution MALDI MSI in positive reflector ion mode. MALDI MSI analysis of porcine ovary section acquired at 22 µm spatial resolution. (**A**) Skyline projection spectrum of molecular species (lipids) in the m/z 200–1200 range; (**B**) Histological image of ovary section (digital scan at 7200 dpi) overlaid (merge) on molecular reconstruction image (Lipids MSI); (**C**) Molecular images of three lipids showing preferential localization to follicular fluid (m/z 578.7), to follicular wall cells (m/z 618.3) and to interstitial tissue outside follicular cells (m/z 704.9).

MALDI MSI analysis of ovarian sections in positive reflector ion mode allowed the visualization of both molecular protonated species and salt adducts (either sodium [M+Na]^+^ or potassium [M+K]^+^) generated from the different lipids, similar to reports from other studies [34,35,36]. The skyline projection spectra generated from all the detected ion signals in the 200–1200 mass range within the analyzed section showed numerous peaks with variable intensity mainly over the 300–900 m/z range (Figure 2A). Such spectra were recorded for more than 120,000 positions throughout an area of ovary section. The ion signal profiles were separated using hierarchical cluster analysis. The most specific clusters were selected to generate representative molecular reconstructions of ovarian tissue sections (Figure 2B).

The multiplexing of specific lipid profiles clearly outlined the differences in lipid composition between the follicles and follicle-free space (named interstitial tissue). Moreover, such differences in lipid distribution were also observed between different follicles. The overlay of the histological digital image and the MSI image showed that lipid profiles varied among ovarian compartments. Indeed, numerous ion density maps demonstrated specific localization and variable abundance of several lipid species inside the ovarian structures (as examples, images for m/z 578.7, 618.3 and 704.9 are presented in Figure 2C). Thus, m/z 578.7 was more abundant in follicular fluid, whereas m/z 618.3 was more frequently represented in follicular wall cells (likely granulosa and theca cells), and m/z 704.9 seemed to be more enriched in the interstitial tissue (Figure 2C).

**Figure 3:**
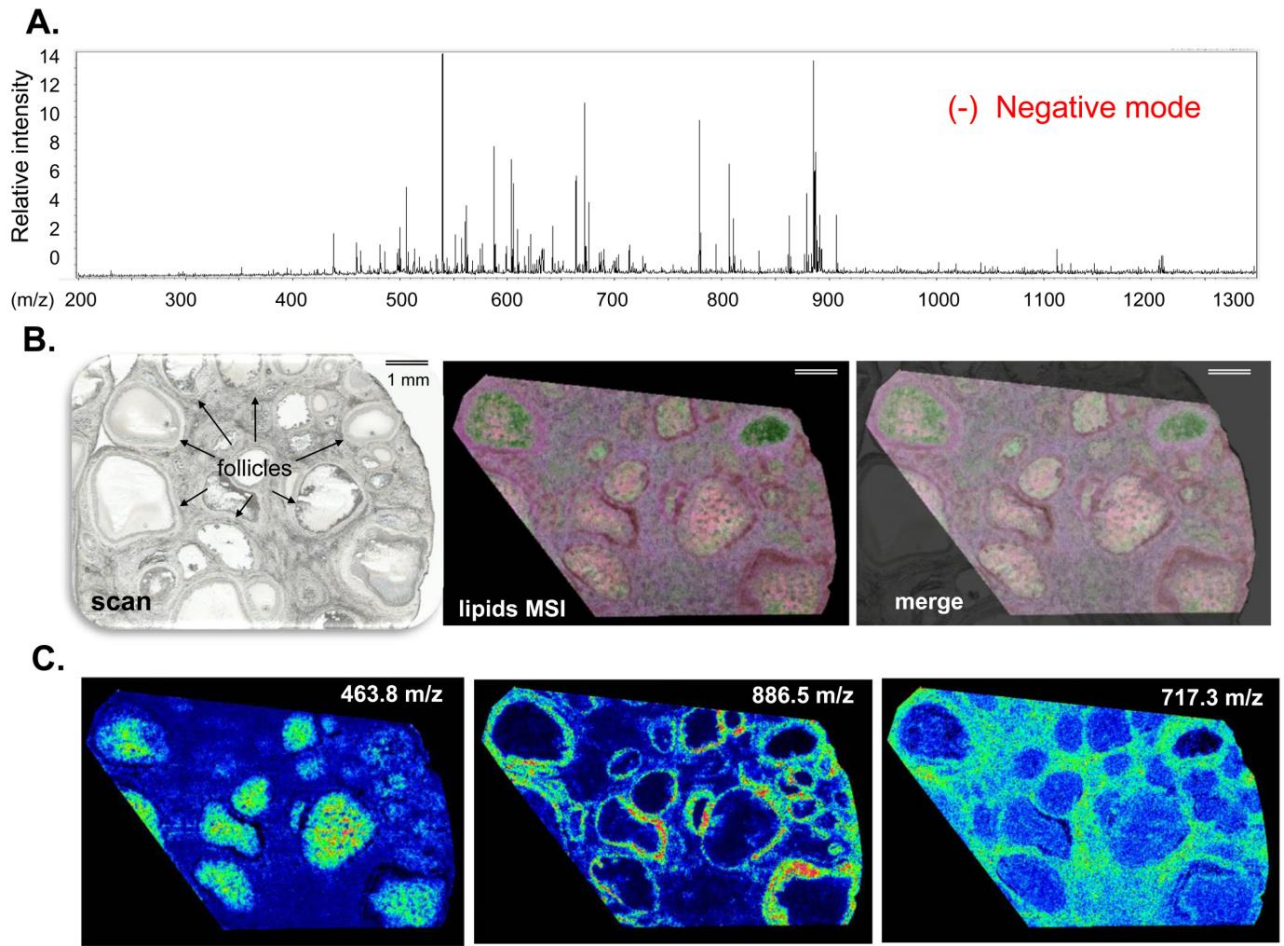
High-resolution MALDI MSI in negative reflector ion mode. MALDI MSI analysis of porcine ovary section acquired at 22 µm spatial resolution. (**A**) Skyline projection spectrum of molecular species (lipids) in the m/z 200–1200 range; (**B**) Histological image of ovary section (digital scan at 7200 dpi) overlaid (merge) on molecular reconstruction image (Lipids MSI); (**C**) Molecular images of three lipids showing preferential localization to follicular fluid (m/z 463.8), to follicular wall cells (m/z 886.5) and to interstitial tissue, mainly outside follicular fluid (m/z 704.9).

MALDI MSI analysis of the same porcine ovary sections was also performed in negative reflector ion mode in the 200–1200 mass range, and enabled visualization of negatively charged ions ([M-H]^−^). In this study, an area overlapping with the positive ion mode analysis was defined to record 98,016 positions. As in positive ion mode, the skyline projection spectra showed numerous peaks with variable intensity mainly in the 450–940 m/z range (Figure 3A). After hierarchical cluster analysis, specific cluster groups were selected to generate a representative molecular reconstruction of the ovarian section (Figure 3B). Multiplex molecular profiles overlaid with the histological digital image outlined characteristic ovarian structures and follicular compartments (FF, OCC, GC, Th). In addition, numerous ion density maps (in Figure 3C, density maps at m/z 463.8, 886.5 and 717.3) showed preferential localization of distinct lipids to different follicular compartments or their variable abundance between the follicles (Figure 3C).

The intensities of specific ions and global lipid profiles, in both positive and negative mode, showed the difference in lipid content between the individual follicles. Such specific spatial distribution patterns may reflect the metabolic difference between follicular cell types and/or fluid contents. Similarly, MALDI MSI lipid profiles differed between cancer and normal tissues in breast, lung, colorectal, esophageal, gastric, and thyroid cancer [37] and in ovarian tumors [29]. Similar to tumors, follicular metabolism is very diverse and is involved in energy production, redox potential and anabolism, notably to support growth [2]. By using specific assays, the concentrations of many metabolites, including non-esterified FA and cholesterol, were shown to be different in follicles of different sizes in cattle [38]. Similarly, ten FAs in FF of women with poor ovarian response to super-ovulation treatment differed compared to women with a normal response [23]. Oocyte competence was also correlated with FA composition in FF in humans [31,39]. Therefore, the differences in lipid content between the different ovarian follicles observed here by MALDI MSI may be due to differences in their stages of folliculogenesis and thus may reflect the different capacities of the follicles to respond to hormonal signals and consequently to their capacity for further maturation.

Approximately 40 lipid ions detected by MALDI MSI using the CHCA matrix were precisely identified by tandem MS/MS analysis in human bone marrow mesenchymal stem cells [40]. These lipid species were detected in similar experimental conditions to ours and were identified as free FAs in the 300–350 m/z range, fragments from phosphatidylcholines (PC) over the 470–670 m/z range, sphingomyelins (SM) over the 682–742 m/z range, phosphatidylethanolamines (PE), phosphatidylglycerol (PG) and PC in the 750–833 m/z range, and phosphatidylinositols (PI) at m/z > 883). In another study, numerous lipid species were noted in porcine oocytes; peaks within 250–340 m/z range were attributed to free FAs and those over the 511–610 m/z range were identified as FA dimers [21]. Phosphatidylserines (PS), PG and PI were ranged between 788 and 888 m/z; m/z 725.43 and 751.44 were identified as diacylglycerols (DAGs) 36:2 and 38:4, respectively, and peaks at m/z > 909 were attributed to TAGs [21]. Moreover, SM, PC and TAG have also been detected in mammalian oocytes by MALDI MS fingerprinting [7,41].

In our study, MALDI MSI analysis of porcine ovary sections also revealed numerous ion species, with most of the peaks ranged between 400 and 900 m/z, several peaks > 900 m/z and several peaks < 400 m/z. Therefore it is possible that among the detected species there could be FA dimers, SMs, PCs, PEs, PIs, mono-, di-, and tri-glycerolipids which might explain the different spatial distribution patterns between the ovarian structures and between the follicles. Tentative identifications of several m/z peaks here detected in positive and negative ion mode by MALDI MSI, are shown in Supplemental data S1. However, the MS/MS approach is required to confirm these attributions.

#### 3.1.2. Intrafollicular Lipid Distribution by MALDI MSI

**Figure 4:**
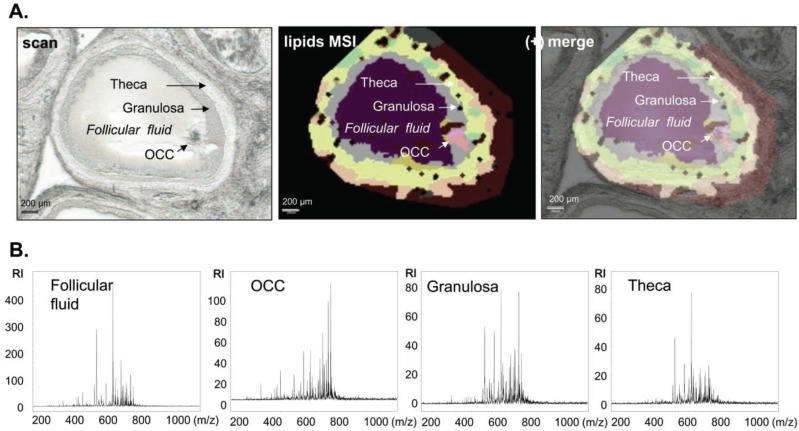
Assessing spatial lipid distribution of the whole follicle from targeted MALDI MSI analysis performed in positive ion mode. (**A**) Histological image of follicle describing the follicular compartments (scan) and its superposition (merge) with molecular reconstruction image (lipids MSI). Scale bars = 200 µm; (**B**) Representative MALDI-TOF MS single spectra acquired directly from the region of interest (Follicular fluid, oocyte-cumulus complex, granulosa and theca) of porcine ovary section in the m/z 200–1000 range. RI = relative intensity.

**Figure 5:**
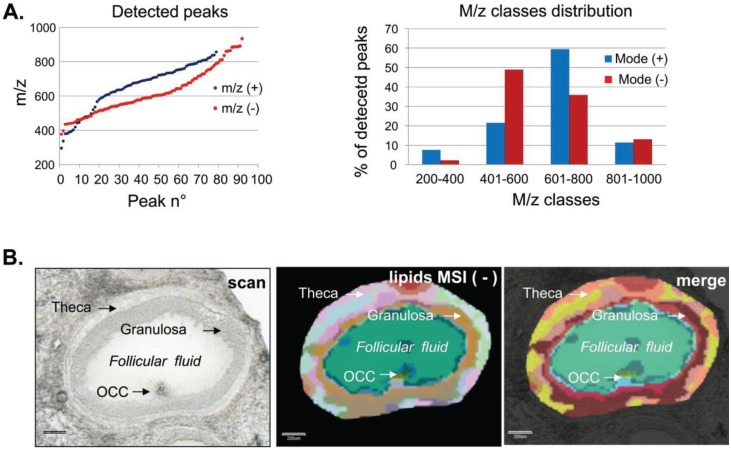
(**A**) Comparative distribution of molecular weight (m/z) of the differential lipid species (ANOVA, *p* < 0.001) detected in positive (blue) and negative (red) reflector ion mode (left panel) and m/z class distributions (right panel). (**B**) Histological image of the follicle describing the follicular compartments (scan), molecular reconstruction image (lipids MSI) and their superposition (merge). Scale bars = 200 µm.

MALDI MSI analysis was targeted at follicles to characterize intrafollicular lipid distribution. The two MSI datasets for positive and negative ion modes were used for differential lipidomic analysis (Figure 4 and Figure 5). Twelve spectra per tissue were extracted from the different follicular compartments including FF, OCC, GC and Th, which were determined by the special morphology of each cell type (Figure 4A,B and Figure 5B). From these spectra profiles, 79 m/z peaks in positive mode and 92 m/z peaks in negative mode were detected at a level 5-fold higher than background. These m/z peaks constituted the basal molecular phenotype of a follicle in each ion mode (Supplemental data, S1).

Lipid ions detected in positive mode presented more peaks in a lower (m/z 200–400) or higher mass range (particularly in 601–800 m/z class) than those detected in negative mode (Figure 5A). This may be explained by the presence of multiple salt adducts in positive mode (masses of 22 Da (Na^+^) or 38 Da (K^+^) and more). In negative mode, more m/z peaks were present in a mid mass range (m/z 401–600) however in a higher mass range (m/z 801–1000) a similar number of peaks were detected in positive and negative modes. Absence of salt adducts in negative mode allowed better detection of molecular species in a wide mass range (92 m/z peaks in negative mode *vs.* 79 m/z peaks in positive, respectively) due to an enhanced signal-to-noise ratio and to a difference in the heterogeneity of the sample modifying the ion suppression effect.

We did quantitative analyses of the spectra extracted from either FF or OCC, or GC or theca in the representative follicles, for both positive and negative ion mode datasets to confirm the visual differences in spatial distribution of lipid species between the follicular compartments. For each m/z peak, the variations in signal intensities between each compartment were compared (Supplemental data, S1). Peaks at 35 and 59 m/z, detected in positive and negative ion mode, respectively, varied between at least two different compartments (*p* < 0.001) more than two-fold. For positive and negative ion mode, respectively, the pairwise comparisons identified 19 and 36 differential m/z peaks between OCC and FF, 4 and 1 differential m/z peaks between theca and GC, 7 and 22 m/z peaks differed between OCC and GC, and 21 and 38 m/z peaks differed between FF and GC (*p* < 0.0001, all differences were more than two-fold).

**Figure 6:**
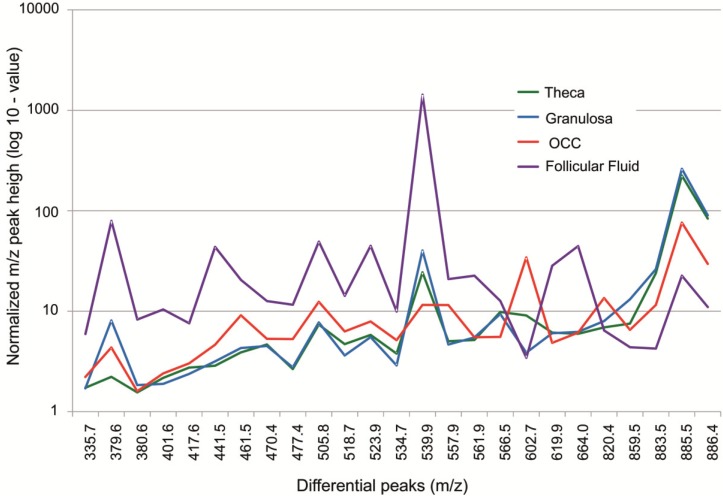
Comparative analysis of the 25 lipid species that differed most among follicular compartments detected by MALDI MSI in positive and negative ion mode. Log-values of normalized peak heights of the ions detected in follicular fluid, theca, granulosa and oocyte-cumulus complex (OCC) of individual follicles are shown.

The top 25 m/z differential peaks detected by MALDI MSI in positive and negative ion modes are shown in Figure 6. Lipid ions measured at m/z 379.6 and 441.5 were approximately 10-fold more abundant in FF than in theca, GC or OCC. The normalized peak height of the lipid ions measured at m/z 461.5 was 4-fold higher in FF than in theca and GC, and 2-fold higher than in OCC. Moreover, the lipid species measured at m/z 602.7 were 10-fold more abundant in OOC compared to GC and FF and 3.5-fold higher compared to theca cells. In contrast, the ions measured at m/z 885.5 were present in both theca and GC at 10-fold higher level than in FF and 3-fold higher than in OCC.

**Figure 7:**
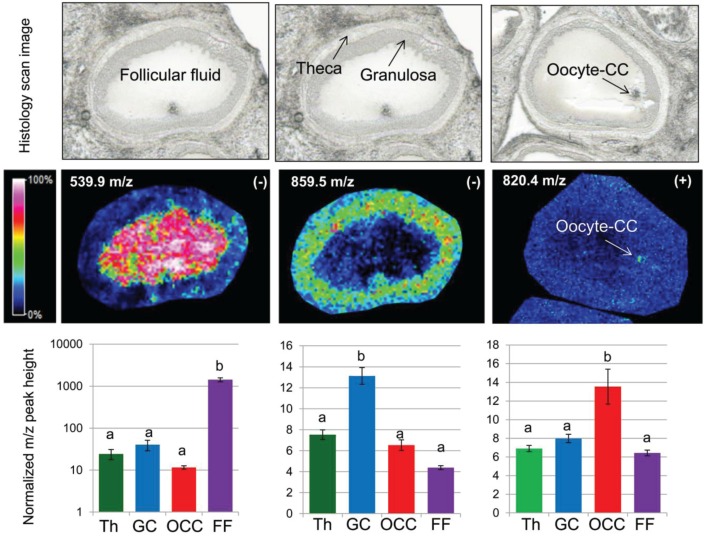
Single ion intensity maps and quantification of three lipid species measured at m/z 539.9, m/z 859.5 (in negative ion mode) and at m/z 820.4 (in positive ion mode), which showed their greater abundance in different follicular compartments—follicular fluid (FF), granulosa cells (GC) and oocyte-cumulus complex (OCC)—of individual follicles. Histograms present the mean values ± standard errors (SEM) of the ion intensities (normalized peak height) measured at 12 positions throughout theca, GC, OCC and FF compartments.

This differential analysis based on quantitative MSI profiles corroborates the images of ion density maps of differential m/z species (Figure 7). The lipid species recorded in negative ion mode at 539.9 and 859.5 m/z showed variability in intrafollicular distribution, with a specific localization in FF and GC, respectively. The intensity of the lipid ion observed in positive ion mode at 820.4 m/z was two times greater inside OCC than in other tissues, most likely corresponding to the oocyte (Figure 7, arrow pointing at OCC).

Specific and very distinct lipid profiles of FF in comparison with follicular cells may be explained by the plasma origin of FF. In fact, FF and plasma have relatively similar biochemical content while only 16 of about 500 proteins detected by 2D SDS-PAGE were different between FF and plasma in humans [42]. Although differences exist between FF and serum composition in mammals [43], plasma levels of most of the metabolites correspond with those of FF; moreover, metabolic changes in serum were reflected by similar changes in FF in cows [44].

The oocyte is a particular cell, the biggest in the follicle. In pigs, as in some other farm species, the oocyte accumulates a large quantity of lipid droplets along with follicular growth [45]. TAGs enclosed in lipid droplets are the main component of porcine oocyte lipids [17,18]. Such lipid composition likely differentiates the oocyte from the other cells, in which accumulation of the TAGs is related to protection against lipotoxity [46].

Only some differences in lipid profiles were found between granulosa and theca cells by MALDI MSI. This finding corroborates active bidirectional cell interactions between these two types of steroidogenic follicular wall cells, which are involved in the control of hormone-producing activity and cell growth [47].

Although this study did not identify particular lipid species, the differences observed between the ovarian compartments may be due to the relative abundance variation of FA dimers, different PC, PI and SM, cholesterol derivatives and glycerolipids (Supplemental data, S1) in accordance with other studies’ identification of lipid m/z species in bovine, porcine and human oocytes, CC and FF [7,20,21,31,48]. According to the relative intensities of different m/z peaks, we conclude that in gilt ovaries, FF and OCC compartments were enriched with low molecular weight species, which may include FA dimers. In contrast, significantly higher abundance of lipids with m/z > 800 occurred in GC, theca and OCC. These ions may be derivatives of PCs, PIs, SMs or di- or tri-glycerols. However, for exact identification of these lipids, secondary high-resolution tandem MS remains essential.

### 3.2. Expression of Lipid Metabolism Genes Varied according to Follicular Compartments

**Figure 8:**
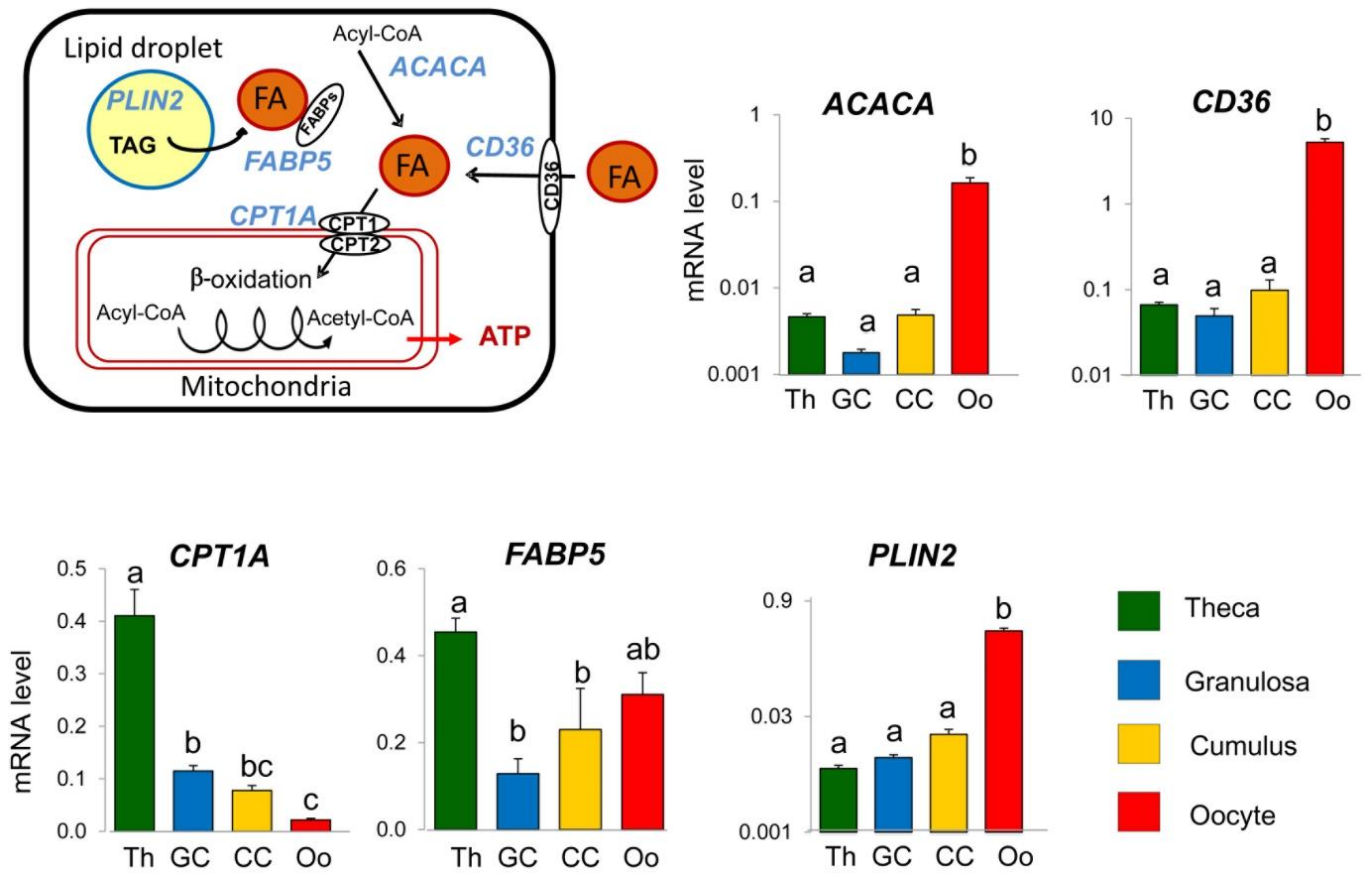
Gene expression analysis of lipid metabolism-related genes *ACACA*, *CD36*, *CPTA1*, *FABP5* and *PLIN2* in porcine follicular compartments (Th, GC, CC and oocyte) by real time qPCR. Histograms present mRNA expression values ± SEM of 8–12 independent samples per compartment.

In order to analyze whether the spatial distribution of lipids in different follicular cell types was supported by differential expression of lipid metabolism-related genes, the mRNA levels of five genes involved in several process of lipid metabolism were compared among cellular follicular compartments, notably GC, theca, oocyte and CC (Figure 8). Using real time PCR quantification, we showed that *ACACA*, *CD36* and *PLIN2* were significantly more expressed in the oocyte than in other follicular compartments, most dramatically compared to CC (33-fold, 53-fold and 20-fold, respectively, *p* < 0.05). This suggests that the oocyte’s potential lipogenic activity is greater than in surrounding tissues. These differences in expression correspond with the functional differences of follicular cells. Indeed, an oocyte has a specific lipid metabolism compared to ovarian somatic cells. In sheep, differences in expression of several genes related to lipid metabolism were found by comparing transcriptomics of the microdissected GC layer and the oocytes from the primordial follicles [49].

Oocyte developmental competence depends in part on the capacity to increase intracellular energy storage because the early embryo cleavages rely on stored lipid [3,13]. To increase its lipid stock, the oocyte may increase *ACACA* expression and thus FA synthesis. *ACACA* uses a substrate, acetyl-CoA, to produce malonyl-CoA which in turn is used by FA synthase as a substrate. *CD36* is involved in FA entry to the cell [50], and by increasing its expression, the entry of FAs into the oocyte may be facilitated, therefore increasing lipid stock, notably through formation of lipid droplets which are mainly composed of TAGs. *PLIN2* interacts with lipid droplets and is involved in maintaining their structure and function [51]. TAGs may serve as a supplemental fuel reserve to support the first embryo cleavages. In addition, by being involved directly in FA synthesis, promoting FA entry and maintaining its lipid droplet structure, the oocyte may reduce FA utilization in two ways. Indeed, according to our data, *CPT1A* expression was significantly lower in the oocyte compared to somatic follicular cells (GC, CC, theca). *CPT1* is responsible for FA entry into mitochondria and is thus essential for FA β-oxidation (for a review, see [52]). By reducing *CPT1A* expression and thus FA β-oxidation, the oocyte may block the use of FA. In addition, *ACACA* is also able to regulate FA β-oxidation. Indeed, the malonyl-CoA produced by *ACACA* is a physiological inhibitor of *CPT1* activity [53]. The oocyte is thus protecting its lipid stores by both reducing *CPT1A* expression at the same time as it enhances lipogenesis.

Theca cells also have a specific function in molecular regulation of lipid metabolism compared to other ovarian compartments. *CPT1A* was significantly more expressed in theca than in GC (by 3.5-fold), and 5-fold more expressed in GC than in the oocyte (*p* < 0.05). *FABP5* was also 3.5-fold more expressed in theca than in GC (*p* < 0.05). In the small immature follicles we used in this study, theca cells were likely less involved in FA synthesis or in saving energy but more involved in lipid storage to provide ATP to the cell for energy consuming functions such as steroid production and cell proliferation. Theca cells could increase energy production by increasing both substrate for FA β-oxidation and FA entry into mitochondria. Indeed, an increase in expression of *FABP5*, the protein that is involved in FA transport and in lipolysis [54,55], may lead to a decrease in the amount of TAGs and to a concomitant increase in free FAs in the cell. Moreover, by increasing *CPT1A* expression, the entry of FAs into mitochondria may also be increased, thus leading to a higher level of production of ATP.

These differences in expression of lipid metabolism genes were consistent with the variations of the lipid profiles between GC, theca and OCC observed in this study using MALDI MSI. In general, an oocyte seems to be lipogenic because of the elevated abundance of free FAs, phospholipids and TAGs as identified by MALDI- and DESI-MS in porcine and bovine oocytes [21,48,56]. This corroborates the high transcript level of *ACACA* and *PLIN2* that we detected in the oocytes. However, the presence of lipolytic activity in the oocytes could be also suggested. Thus, CPT1 protein and hormone-sensitive lipase were detected in bovine immature oocytes at a higher level than in CC [12,57]. According to the higher expression of *CPT1A* and *FABP5*, theca cells seem more lipolytic than GC and CC. The difference in GC and theca lipid metabolism was also reported in one study where the same treatment with polyunsaturated FAs affected steroidogenesis only in theca cells rather than in GC in sheep [58]. In addition, the particular lipid content of FF may be due both to different metabolic activities of follicular cells and to external direct delivery of FAs from blood to FF.

## 4. Conclusions

In the present study, we were able to map numerous lipid species to specific cell types in porcine ovaries by MALDI MSI and to reconstruct ovarian section morphology using lipid ion signals measured in both positive and negative ion modes. Different ovarian structures showed specific lipid patterns; in particular the follicles were quite different from non-follicular ovarian tissues. Moreover, according to reconstructed lipid MSI images, the heterogeneity of lipid composition was also observed between different follicles, underlining the individuality of the metabolome of each follicle which may reflect properties of the follicular environment for oocyte development. Inside the follicles, lipids were also distributed differently among intrafollicular compartments. Different cell layers, notably granulosa, theca and oocyte-cumulus complex, had lipid compositions that differed from those of inter follicular fluids. These differences in lipid composition between the follicular compartments were supported by a gene expression study that underlined the particular importance of the different steps of FA metabolism in different follicular cells and allowed better understanding of their specific functions. We conclude that lipid metabolism occurs in all the follicular cellular compartments, with possible particular involvement of the theca cells in FA oxidation. The enrichment of some specific lipids in the oocyte and FF needs to be studied in more detail. Further analysis including a high-resolution MS in combination with tandem MS are necessary for accurate identification of the lipid species in the ovarian tissues.

## Supplementary Files

Supplementary File 1
